# Genome-wide association study for *posthitis* in the free-living population of European bison (*Bison bonasus*)

**DOI:** 10.1186/s13062-014-0033-6

**Published:** 2015-01-14

**Authors:** Kamil Oleński, Małgorzata Tokarska, Dorota Maria Hering, Paulina Puckowska, Anna Ruść, Cino Pertoldi, Stanisław Kamiński

**Affiliations:** Department of Animal Genetics, University of Warmia and Mazury in Olsztyn, 10-718 Olsztyn, Poland; Mammal Research Institute Polish Academy of Sciences, Białowieża, Poland; Department 18/Section of Environmental Engineering, Aalborg University, Sohngårdsholmvej 57, 9000 Aalborg, Denmark; Aalborg Zoo, Mølleparkvej 63, 9000 Aalborg, Denmark

**Keywords:** Bison, *Posthitis*, GWAS, Illumina BovineHD 777K

## Abstract

**Background:**

About 5–6% of the European bison (*Bison bonasus*) males are affected by *posthitis* (necrotic inflammation of the prepuce) and die in the wild forest. Despite many years of study, pathogenesis of this disease has not yet been determined. The main aim of the study was to find SNP markers significantly associated with the incidence of *posthitis* and mine the genome for candidate genes potentially involved in the development of the disease.

**Results:**

It was shown that relatively small number of SNPs effects reached genome-wide significance after false discovery rate (FDR) correction. Among 25 significant markers, the highest effects were found for two SNPs (rs110456748 and rs136792896) located at the distance of 23846 bp and 37742 bp, respectively, from *OR10A3* gene (olfactory receptor genes), known to be involved in *atopic dermatitis* in humans. It was also observed that five other significant SNP markers were located in the proximity of candidate genes involved in severe diseases of skin tissue and cancer/tumour development of epithelial or testicular germ cells, which suggest their potential participation in the *posthitis*. The 25 investigated SNPs showed marked differences in allelic and genotypic frequencies between the healthy and affected bison groups.

**Conclusions:**

The 2 Mb region of the BTA15 chromosome is involved in genetic background of *posthitis* and should be closer examined to find causal mutations helpful in better understanding of the disease ethology and to control its incidence in the future.

**Reviewers:**

This article was reviewed by Prof. Lev Klebanov and Dr. Fyodor Kondrashov.

## Background

Genome Wide Association Studies (GWAS) have become one of the major tools in explaining the ethology of human diseases of genetic origin [1]. The technique has also been applied in studies on model organisms [2] and domestic animals [3,4] as well as in crops [5]. Performing GWAS in wild living species is usually difficult, as all the commercially available microarray sets are designed for model or domestic animals. It has been shown that cattle SNP microarrays work well in bison species [6-8]. The European Bison (*Bison bonasus*), (EB), after extinction in the wild before World War II, was restored based on progeny of just 12 individuals preserved in private collections and zoological gardens [9]. The restored population was divided into two isolated genetic lines: the Lowland or the Bialowieza line (in which the founders were seven pure Lowland bison) and the Lowland-Caucasian line (in which the founders were 11 Lowland bison individuals and one Caucasian bison bull). Both populations of the EB are extremely inbred [10].

In the last four decades, a severe disease called *posthitis* affected EB males. This disease was discovered in the 1980s in the Bialowieza Forest [11,12]. At the end of the 1990s, similar symptoms were also observed in five young EB of the Lowland line from the Bayerisher Wald National Park, Germany [13]. *Posthitis* is defined as necrotic inflammation of the prepuce. About 5–6% of the males are affected each year and die in the wild forest or are eliminated by officially approved hunting [14]. Despite many years of study, pathogenesis of *posthitis* in bison has not yet been determined. The affected bulls do not exhibit changes in the general physiological mechanism as indicated in the long-term studies of 30 physiological indices [15]. *Posthitis* is thought to be a disease of an endemic character since it is only observed in males living in Bialowieza National Park or transported from it. Apparently, no environmental or genetic factors which would explain the susceptibility of some individuals to develop the disease have been identified [14]. New tools for whole genome analysis (SNP microarrays) available for the Bovidae species since 2009 (www.illumina.com) have opened new possibilities to improve the genetic analysis of *posthitis*.

Among the commercially available microarrays, the Illumina Bovine HD 777 K microarray is the most suitable tool for bison genome studies. Its usefulness was evaluated recently by showing that the Lowland line is a genetically homogenous population, with only a small amount of polymorphic loci, which can be helpful in pedigree analysis [7,16]. It was also shown that only 2.8% out of 54,000 bovine point mutations are polymorphic in ten EB representing the Caucasian and Lowland lines [8].

In this paper, we used high density bovine SNP microarray to investigate the entire bison genome in order to identify differences in SNP allele and genotype frequencies between the group of individuals affected by *posthitis* and the non-affected group. The differences in allelic frequency found between these two groups were used to perform a GWAS to identify genes potentially involved in the *posthitis* disease.

## Results

**Figure 1:**
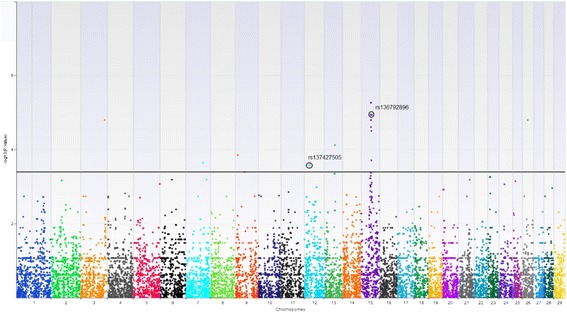
**Results of the statistical analysis.** Manhattan plot showing global distribution of 18,079 SNPs along the genome of bison to map SNP associated with *posthitis*. SNPs above the solid line represent a significant association [−log10(P-value) = 3,4]. Two circled markers were sequenced to check whether bovine SNP give the same genotype on bison DNA.

A global view of all SNPs effects for *posthitis* incidence is presented in the Manhattan plot in Figure 1. It is shown that a relatively small number of SNPs effects reached genome-wide significance after FDR correction. Among the 25 top significant markers, most of them (18) were located on chromosome 15. Single significant markers were also found on chromosome 3, 9, 13, 26 and three markers on chromosome 12.

Detailed characterization of the top 25 markers are given in Table 1, including the SNP position on UMD 3.1 genome map, the name of the closest candidate gene and its distance from the significant marker and the reference confirming the potential involvement of a candidate gene in *posthitis*. Eleven significant markers were located on chromosome 15 in the close vicinity of olfactory receptor genes.

**Table 1:** **SNPs with highest effects on the** ***posthitis*** **incidence**

**SNP id**	**Chr**	**SNP position**	**Closest candidate gene**			**Reference**
			**Symbol**	**Distance from SNP marker (bp)**	**Name**	
rs110456748	15	45238956	*OR10A3*	23846	Olfactory receptor, family 10, subfamily A, member 3	[20]
rs136792896	15	45252852	*OR10A3*	37742		
rs109096473	15	45264139	*LOC539064*	46997	Olfactory receptor 1030-like	
rs43150190	15	45271797	*LOC539064*	39339		
rs134156378	15	46596723	*LOC516636*	6473	Olfactory receptor 6-like	
rs42993236	15	47910912	*LOC507428*	1314	Olfactory receptor, family 52, subfamily N, member 2-like	
rs41986233	15	48091239	*LOC787878*	0	Putative olfactory receptor 56B2-like	
rs41986306	15	48143907	*LOC100139556*	3799	Olfactory receptor 56B1-like	
rs42503200	15	45475858	*LOC782645*	2580	Olfactory receptor, family 10, subfamily R, member 2-like	
rs136242790	15	45239543	*OR10A3*	24433	Olfactory receptor, family 10, subfamily A, member 3	
rs109730608	15	46420735	*OR2D3*	1737	Olfactory receptor, family 2, subfamily D, member 3	
rs42505727	15	45714724	*PPFIBP2*	0	PTPRF interacting protein, binding protein 2 (liprin beta 2)	[22]
rs41597707	15	45743620	*PPFIBP2*	0		
rs43674350	15	45773658	*PPFIBP2*	0		
rs43674363	15	45779930	*PPFIBP2*	0		
rs41766565	15	45858786	*OLFML1*	0	Olfactomedin-like 1	[23]
rs133100902	15	45865065	*OLFML1*	0		
rs134495844	15	46365446	*ZNF214*	3184	Zinc finger protein 214	[18]
rs110088444	26	25589629	*SORCS3*	0	Sortilin-related VPS10 domain containing receptor 3	-
rs137458105	13	48582285	*LRRN4*	0	Leucine rich repeat neuronal 4	-
rs134132514	9	10444161	*B3GAT2*	142985	Beta-1,3-glucuronyltransferase 2 (glucuronosyltransferase S)	-
rs137024050	12	22730025	*COG6*	0	Component of oligomeric golgi complex 6	[20]
rs133483310	12	22928947	*LHFP*	0	Lipoma HMGIC fusion partner	[19]
rs137427505	12	23622735	*UFM1*	62366	Ubiquitin-fold modifier 1	-
rs134255411	3	109983658	*CSF3R*	78187	Colony stimulating factor 3 receptor (granulocyte)	[28]

Top 25 SNP markers showing the highest effect associated with the incidence of *posthitis* (with FDR correction) and suggested candidate genes located in the closest vicinity to significant marker.

To better show how the number of SNP genotypes change between affected and unaffected bison, the allelic and genotypic frequencies of the 25 markers were estimated for the two bison groups and are listed in Table 2.

**Table 2:** **Frequency of genotypes and alleles**

**SNP id**	**Number of genotypes**						**Allele frequency**				**Number of missing genotypes**
	**Affected**			**Unaffected**			**Affected**		**Unaffected**		
	***AA***	***AB***	***BB***	***AA***	***AB***	***BB***	***A***	***B***	***A***	***B***	
rs110456748	40	13	1	13	15	8	0.861	0.139	0.569	0.431	0
rs136792896	40	13	1	13	15	8	0.861	0.139	0.569	0.431	0
rs109096473	1	13	40	8	15	13	0.139	0.861	0.431	0.569	0
rs43150190	1	13	40	8	14	13	0.139	0.861	0.429	0.571	1
rs42505727	39	14	1	13	15	8	0.852	0.148	0.569	0.431	0
rs41597707	39	14	1	13	15	8	0.852	0.148	0.569	0.431	0
rs43674350	1	14	39	8	15	13	0.148	0.852	0.431	0.569	0
rs43674363	1	14	39	8	15	13	0.148	0.852	0.431	0.569	0
rs41766565	39	14	1	13	15	8	0.852	0.148	0.569	0.431	0
rs133100902	39	14	1	13	15	8	0.852	0.148	0.569	0.431	0
rs134495844	1	14	39	8	15	13	0.148	0.852	0.431	0.569	0
rs134156378	1	14	39	8	15	13	0.148	0.852	0.431	0.569	0
rs42993236	1	14	39	8	15	13	0.148	0.852	0.431	0.569	0
rs41986233	39	14	1	13	15	8	0.852	0.148	0.569	0.431	0
rs41986306	1	14	39	8	15	13	0.148	0.852	0.431	0.569	0
rs42503200	39	13	1	13	14	8	0.858	0.142	0.571	0.429	2
rs110088444	0	14	33	8	14	12	0.149	0.851	0.441	0.559	9
rs136242790	1	13	40	7	15	13	0.139	0.861	0.471	0.529	1
rs109730608	1	14	39	8	14	13	0.148	0.852	0.429	0.571	1
rs137458105	0	0	50	0	9	26	0.000	1.000	0.129	0.871	5
rs41594613	53	0	0	25	8	0	1.000	0.000	0.879	0.121	4
rs134132514	0	2	52	2	10	24	0.019	0.981	0.194	0.806	0
rs137024050	13	24	17	20	14	2	0.463	0.537	0.778	0.222	0
rs133483310	17	24	13	2	14	20	0.537	0.463	0.222	0.778	0
rs137427505	13	24	17	20	14	2	0.463	0.537	0.778	0.222	0
rs134255411	0	13	33	0	24	12	0.141	0.859	0.333	0.667	8

The frequency of genotypes and alleles in 25 significant SNPs observed in healthy and affected bison.

**Figure 2:**
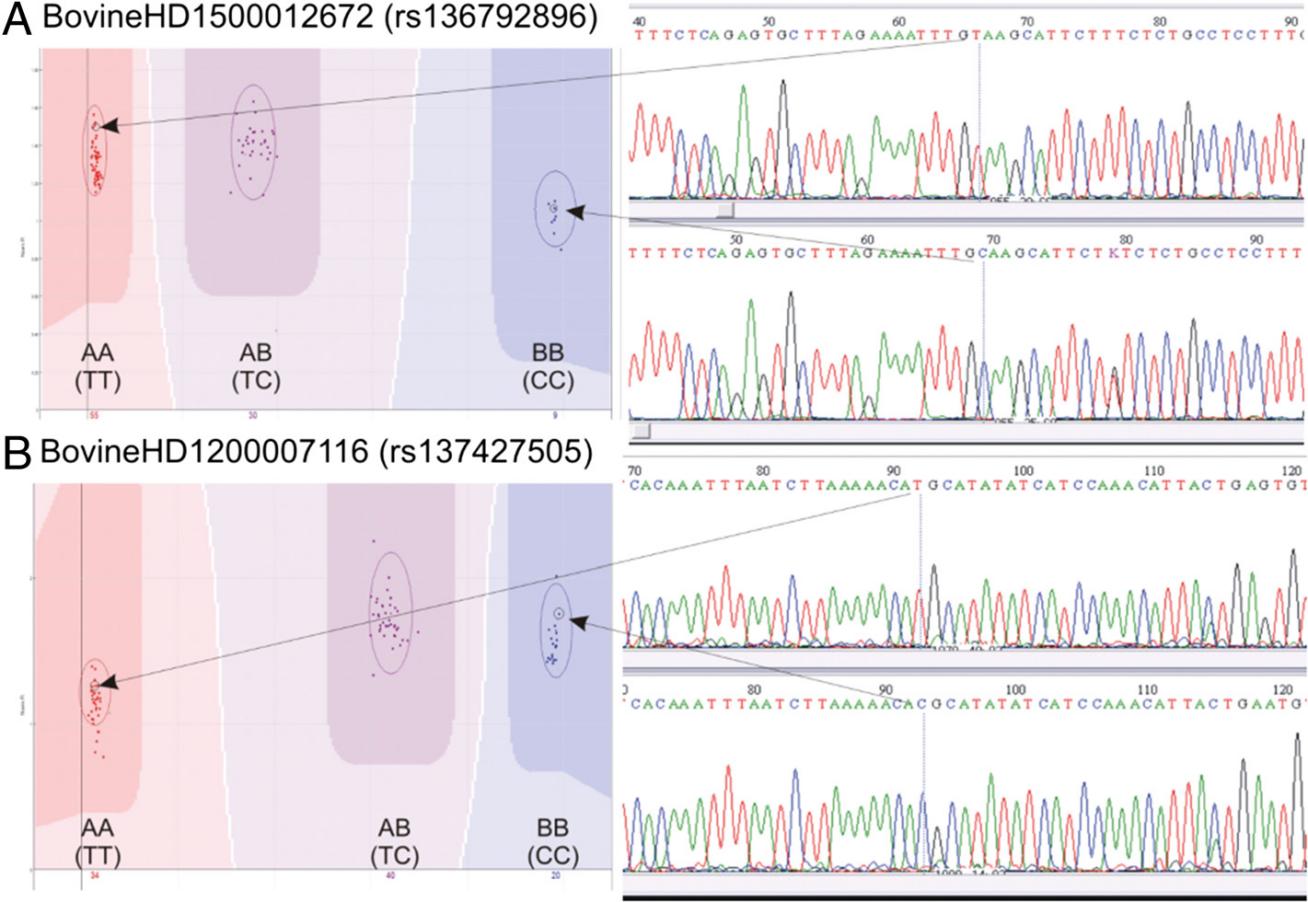
**Correctness of microarray genotyping.** Checking the correctness of genotyping on two randomly-chosen significant SNPs. **A** - SNP rs136792896 located on chromosome 15, **B** - SNP rs137427505 located on chromosome 12. Genotypes designated by Illumina Bead Studio (on the left) agree with the genotypes obtained by sequencing (on the right).

For two randomly chosen significant markers, SNP genotypes designated by Illumina Bead Studio were checked by sequencing (rs136792896and rs137427505). No discrepancies between the alternative genotypes (*AA* vs *BB*) for these SNPs were found (Figure 2).

## Discussion

European bison is an example of an extinct species, which was successfully reintroduced to wild nature. Due to the founder effect, the current free-living bison population of the Lowland line (about 1000 individuals in 2011 [17]) is extremely inbred and, therefore special attention should be paid to any threats decreasing the reproductive potential of the population and, consequently, lowering the genetic diversity of the species [9].

The inbreeding level of EB was also considered as a potential factor increasing the risk of disease, but the difference between the inbreeding coefficient of healthy (32 bison) and affected (12 bison) was not found to be significant [18]. Another analysis of 69 affected males did not find significant associations between MHC DRB3 alleles and the susceptibility to *posthitis* [19].

In this study, the utilization of an HD (High Density) microarray, allowed a detailed screening of 18,079 polymorphic markers which were used in a GWAS analysis to identify those significantly associated with the incidence of *posthitis*. In Figure 1 and Table 1 it is shown that a group of markers located on chromosome 15 have the highest effect. They are located within *OLFML1* and *PPFIBP2* and in the close vicinity of the *ZNF214* gene. Most significant effects were found for two SNPs (rs110456748 and rs136792896) located at a distance of 23846 bp and 37742 bp from *OR10A3* gene, known as being involved in *atopic dermatitis*, a common inflammatory skin disease [20].

Several significant markers were mapped within two genes: *PPFIBP2* and *OLFML1. PPFIBP2* encodes protein-tyrosine phosphatase-interacting protein, which is differentially expressed in endometrial cancer [21] and is also involved in tumour cell migration and invasiveness of extracellular signal-regulated kinase depleted cells [22]. *OLFML1* (olfactomedin-like protein 1) encodes a secreted protein, is expressed in many tissues and may play a significant role in the regulation of cell proliferation *in vitro* [23]. Another gene located closely to significant marker rs134495844 is coding zing finger protein 214 (*ZNF214*) - a potential tumour suppressor gene whose expression is increased in tumours derived from testicular germ cells [24]. The next candidate genes, *COG6* and *LHFP*, were found to be associated with susceptibility to *psoriasis* [25,26]. Among the genes listed in Table 1, five candidate genes are associated with severe skin diseases (*atopic dermatitis, psoriasis*) or cancer/tumour development of epithelial or testicular germ cells, which suggest their potential participation in the *posthitis*.

The fact that the remaining five candidate genes (Table 1) do not show a direct link with an inflammation/necrosis specific function does not exclude the possibility that these genes are contributing to *pothistis* susceptibility. It is possible that their contribution is indirect, through immunological pathways, or has not yet been reported. It can be assumed that, as for any complex trait, many genes whose names are not “trait-specific” participate in the overall genetic variance of the trait [27].

Being aware that in typical GWAS, most of the significant markers are located outside the functional genes and are in linkage disequilibrium with other genes, we additionally used the MapViewer service to identify genes located in the broader neighborhood of the significant marker (at a distance of less than 1 Mb). The size of the inspected region is difficult to be justified objectively since the distribution of the functional gene along the chromosomes is very different and specific to each autosome [28]. One Mb distance from a significant marker seems to be a manageable region to manually screen all genes included. All such genes were checked for functional association with inflammation/tumour/cancer/necrosis. In this way, we were able to find further candidate genes related to the trait of interest. For example, *COL17A1* encoding collagen type XVII, alpha 1 (located 529,5 Kb from the significant marker rs110088444, on BTA 26) is involved in *epidermolysis bullosa*, causing separation of the basal keratinocyte from the underlying basement membrane and consequently skin atrophy [29]. *TRIM6* coding tripartite motif 6 located 3799 bp from the significant marker rs1986306 has been reported as playing a role in an IFN-induced antiviral state against retrovirus infection [30]. *ILK* gene encoding integrin-linked kinase (mapped 477,9 K from significant marker rs134156378, BTA15) interacts with pathogenic bacteria reinforcing host cell adherence [31]. Another gene associated with skin pathology, *FERMT1* encoding fermitin family member 1 is located 34,7Kb from significant marker rs137458105 (BTA13). Its mutation causes Kindler syndrome, whose symptoms include skin blistering, skin atrophy, photosensitivity, colonic inflammation and mucosal stenosis [32]. Proliferating cell nuclear antigen (*PCNA*) is located about 787Kb from marker rs137458105. It was shown that the percentage of *PCNA*-positive cells in malignant and some non-malignant skin diseases (*atopic dermatitis*, *psoriasis*) is from 2- to 5- times higher than in normal skin cells [33].

In the close vicinity of significant marker rs134255411 (BTA 3) *CSF3R* gene encoding colony stimulating factor 3 receptor is located. Its mutation causes chronic neutrophilia [34]. It was reported that periostin, osteoblast specific factor, a protein encoded by the *POSTN* gene exacerbates the pathogenesis of *atopic dermatitis* in mice [35,36]. *POSTN* is located 618Kb from the significant marker rs137427505 on BTA12. Another gene located near the significant marker rs137024050 is forkhead box O1 transcription factor, whose loss of expression in lesional psoriatic tissues has a potential contribution to the development of *psoriasis* [37].

All of the above-mentioned reports support the main outcome of our work – that the most significant markers for *posthitis* indicate regions containing genes involved in the immune system and disorders of skin and epithelial cells. Unlike in Johnston [3], we used marker set of extremely high coverage of the cattle genome – over 770,000 *loci*. Attempts to use standard microchips (of approx. 54,000 SNPs) failed as we could not find any marker associated with the disease symptoms (data not shown). In future research, GWAS should be validated for a larger population of *posthitis*-affected bison and candidate genes should be screened to find causal mutations enabling a better understanding of the disease and, prospectively, to control its incidence. The fact that the 25 SNPs investigated showed marked differences in allelic and genotypic frequencies between the healthy and affected bison groups is a clear indication of an excess of the genotype *BB* in the affected population together with a higher frequency of the *B* allele in the affected compared to the non-affected bison population. In addition, the fact that many alleles showed the same allelic frequency indicate a strong linkage between several SNPs on the same chromosome. This suggests that an appropriate breeding strategy based on identity by descent (IBD) could be developed for the European bison. Information from the SNP chip could be used as an accurate tool for guiding which individuals should mate, and which should be excluded from the mating as they are bearing the alleles associated with incidence of *posthitis*. Given the fact that many of the SNPs which showed associations are strongly linked, it would be enough to simply screen one of the SNPs which are linked to each other. At the same time it would also be desirable to optimize the best contribution of animals to the next generation, in order to reduce the pace at which the inbreeding coefficient is increasing every generation. This can be in the form of a specific list with suggested matings or guidelines on the number of matings that a given bison should be engaged in during a given number of generations. Developing a SNP chip with a subset of SNPs found to be associated with the *posthitis* incidence and with a subset of SNPs informative for paternity analysis and to estimate the kinship between individuals could be useful for this purpose and make these goals more realistic from an economic point of view.

## Conclusions

Allele frequencies of 25 SNP markers are significantly different in health and *posthitis*-affected animals. Genes localized in the closest vicinity of these markers show connections with immunological processes. 18 out of 25 markers are localized on chromosome BTA15 within the space of 2 Mb, which suggests this region may play an important role in the ethology of the *posthitis*. This subregion should be closer examined to find causal mutations helpful in better understanding of the genetic background of the disease and to control its incidence in the future.

## Methods

### Animals

**Figure 3:**
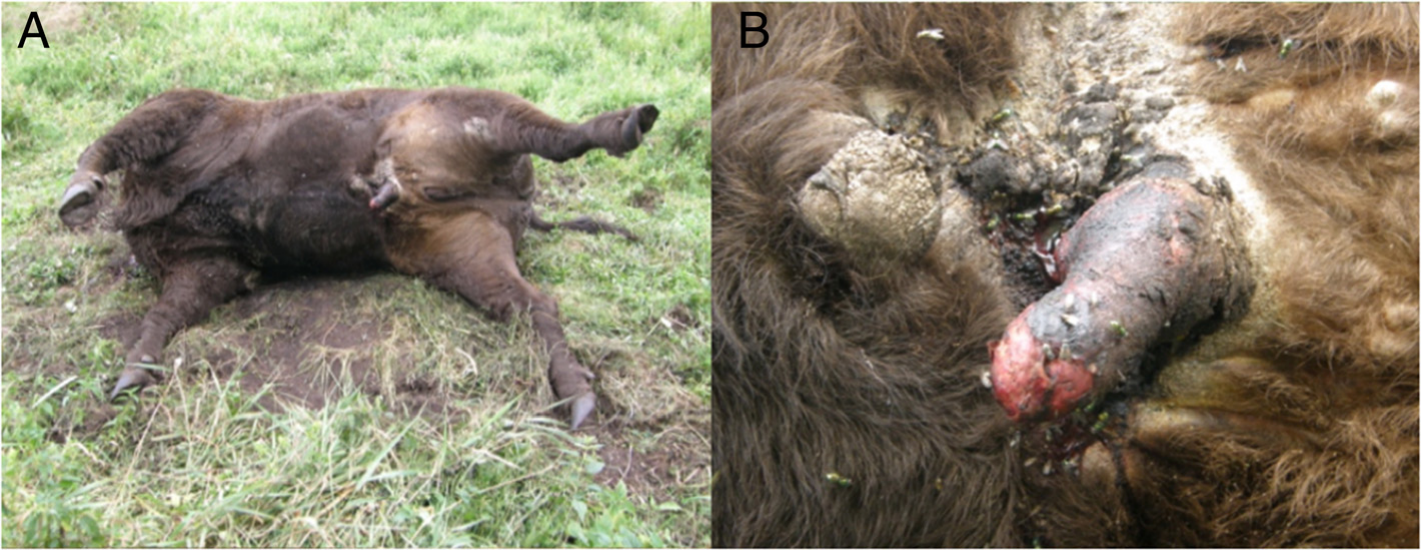
**An example of bison affected by** ***posthitis*** **. A**. Affected bison (Courtesy of Mammal Research Institute in Bialowieza) found in the National Park in Bialowieza. **B**. Magnification of genital area. Necrosis of prepuce is visible as advanced lesions in skin tissue surrounding the penis.

The study included 90 adult males of the Lowland line of the EB living in the Bialowieza National Park. Bison DNA samples were provided by the Mammal Research Institute in Bialowieza (Polish Academy of Sciences). The animals were divided into two groups: healthy (36) and affected by *posthitis* (54). The presence of the disease and the degree of its advancement were ascertained *post mortem* by an expert veterinarian, on the basis of anatomical and pathological changes [38] (Figure 3).

### Genotyping and statistical analysis

Each individual was genotyped using the Illumina BovineHD 777 K microarray, which consists of 777,962 SNP markers. Associations were tested using GoldenHelix SVS7 analysis software (Bozeman, MT). Initial data clean-up was performed to remove poorly performing SNPs. SNPs in the control population with significant deviations from the Hardy–Weinberg equilibrium (HWE) at the significance level of P < 0.0001 were removed prior to association analysis. Additionally, the SNP selection criteria were applied based on a minor allele frequency (MAF) of at least MAF > 0.005, due to highly monomorphic data and the technical quality of the chip – a minimum call rate of 98%. After filtering, 18,079 SNPs were used in the final analysis. The density of SNPs used in the analysis was 1 SNP per 138 kb. The average call rate obtained for our data set amounted to 98.86% (SD = 0.2%) for selected SNPs.

An additive model of the Linear Regression Analysis with a False Discovery Rate, kinship matrix (computed on the autosomal SNPs in SVS7.6) and heterozygosity rate as additional covariate was performed to estimate the effect of an SNP marker for *posthitis* incidence. SNP association analysis was performed on autosomal SNPs. Following this, the allelic and genotypic frequencies of the significant SNP markers were then estimated for the two bison groups.

### Sequencing

Since bison DNA was used on a bovine SNP array, two fragments of bison genomic DNA (containing two SNPs) were amplified and sequenced to check whether the SNP genotype was correct. The following SNPs were PCR-amplified and sequenced: BovineHD1500012672 (rs136792896) and BovineHD1200007116 (rs137427505). Primers were designed and blasted using the NCBI primer-blast website (http://www.ncbi.nlm.nih.gov/tools/primer-blast/):

BOVHD15-L: 5′–GGCCTTCACTGTCCACCTTA–3′

BOVHD15-R: 5′–TTAAATCACTGCCCCCAAAG–3′

BOVHD12-L: 5′–ACCATCACAAGCAGACTGCCCA–3′

BOVHD12-R: 5′–AGCAGAATCGTGAATGTGGCTGGT–3′

In the PCR reaction, 20 pM of each primer, 1 U of Taq DNA polymerase (Bioron, Germany), 100 ng of genomic DNA, 2.5 μl of 10× PCR buffer, 2.5 mM of MgCl_2_ and 800 μM of dNTPs were used. The amplification program consisted of: an initial DNA denaturation (94°C/3 min), 35 cycles of denaturation (94°C/30 s), annealing (61°C/30 s) and elongation (72°C/30 s), final synthesis (72°C/5 min).

PCR yielded two specific amplicons of expected 185 (BovineHD1500012672) and 537 bp (BovineHD1200007116). The PCR products were purified and sequenced by the GENOMED Ltd (Poland). The sequences were analyzed using BioEdit v. 7.2.0 software.

### Mining genome for candidate genes

Two approaches were used to locate candidate genes: statistical and functional. In the statistical approach, genes located in the closest vicinity of markers above the cut-off line were chosen (P value after FDR correction was 3.40). In the functional approach, 1 Mb region around the position of the significant SNP marker was inspected in the MapViewer service (www.ncbi.nlm.nih.gov) in order to check whether there are genes functionally associated with inflammation or diseases showing symptoms related to *posthitis*.

The genomic positions of SNPs included in the Illumina BovineHD 777 K were taken from the Illumina publication (www.illumina.com). Genomic positions of candidate genes were assigned based on the UMD 3.1 bovine genome assembly [39] through the current Ensembl database (www.ensembl.org).

### Statement of ethical approval

Tissue samples were collected during annual culling of the European bison approved by General Directorate for Environmental Protection (Warsaw, Poland) and Regional Directorate for Environmental Protection (Bialystok, Poland) and released for Mammal Research Institute in Bialowieza. Reference numbers of the approvals: 2010: DOP-Pozgiz-4200/IV-57/4139/10/kp; 2011: DOP-OZGTZ.6401.05.5.2012.kp; 2012: WPN.6401.222.2012.EJ; 2013: WPN.6401.266.2013.EJ; 2014: WPN.6401.221.2014.EJ.

## Reviewer comments

We appreciate very much all remarks, suggestions and comments provided by reviewers. We considered them carefully trying to improve the manuscript.

### Reviewer 1: Dr. Fyodor Kondrashov

I do not see any issues in the design and implementation of the present study and the results are straightforward. The authors capitalized on the application of tools that have been developed for domestic species to one of conservation value. The study identifies several candidate genes for a specific disease, *posthitis*, which can now drive a more enlightened breeding effort to reduce its impact. While this study is a good demonstration of how tools developed for specific industries can be taken advantage of for environmental issues, it is unlikely that species without a closely related relative of economic impact can be studied in a similar manner.

Quality of written English: Acceptable

Author respond: We appreciate very much sharing our view on how new tools developed for domestic animals may be efficiently used for related species living in wild conditions.

### Reviewer 2: Prof. Lev Klebanov

The manuscript contains an interesting study of finding SNP markers significantly associated with the incidence of *posthitis* and mine the genome for candidate genes potentially involved in the development of the disease. The methods developed here are based on the use of high-density bovine SNP microarray to investigate the entire bison genome. The study shows that among the 25 top significant markers, 18 were located on chromosome 15. Eleven significant markers were located on chromosome 15 in the close vicinity of olfactory receptor genes. In my opinion, the manuscript on the review is very interesting and worth to be published in Biology Direct. However, I have some questions, and think that the answers them may make the presentation better. Namely, on page 10 there is written: “SNPs with significant deviations from the Hardy–Weinberg equilibrium (HWE) at the significance level of P < 0.0001 were removed prior to association analysis. Additionally, the SNP selection criteria were applied based on a minor allele frequency (MAF) of at least MAF > 0.005, due to highly monomorphic data and the technical quality of the chip – a minimum call rate of 98%.” I did not understood, why such levels of P and MAF were used here.

Author respond: Illumina BovineHD BeadChip is designed especially for the domestic cattle and some out-group *Bovidae* species (bison, water buffalo, yak). The out-group species are only related with *Bos* species, therefore we have to be aware that bovine SNPs sometimes may generate spurious results when we use bison DNA. The only way to minimize that is to exclude all samples (bisons) giving low call rate. Moreover careful analysis of SNP cluster quality is also of key value. It was the reason why we used higher cal rate (>98%) than standard value (95%).

Another problem with bison SNPs is their limited number. The Lowland (Bialowieza) line of the European bison was restored from 7 pure Lowland animals – one male and six females. The relationship and the inbred level of all living animals are extremely high. Obviously, such low heterozygosity reduces the number of SNPs available for association study. Our intention was to keep almost each polymorphic SNP to cover the whole genome with markers located with relatively short distance from each other. Therefore we decreased the typical level of MAF from 0.01 to 0.005.

Summing up, both criteria were used to maximize the number of good-quality heterozygotic SNPs.

Analyzing criteria for SNP selection we found that one sentence should be corrected “SNPs in the control population with significant deviations from the Hardy–Weinberg equilibrium (HWE) at the significance level of P < 0.0001 were removed prior to association analysis”.

## Abbreviations

EB: Eurpoean bison
GWAS: Genome-Wide Association Study
IBD: Identity By Descent
HD: High Density
BTA: Bos Taurus Autosome
FDR: False Discovery Rate
SNP: Single Nucleotide Polymorphism
MAF: Minor Allele Frequency
Mb: Megabases (10^6^ basepairs)
kb: kilobases (10^3^ basepairs)
HWE: Hardy-Weinberg Equilibrium
UMD: University of Maryland Database
